# A three-dimensional culture system for generating cardiac spheroids composed of cardiomyocytes, endothelial cells, smooth-muscle cells, and cardiac fibroblasts derived from human induced-pluripotent stem cells

**DOI:** 10.3389/fbioe.2022.908848

**Published:** 2022-07-22

**Authors:** Asher Kahn-Krell, Danielle Pretorius, Bijay Guragain, Xi Lou, Yuhua Wei, Jianhua Zhang, Aijun Qiao, Yuji Nakada, Timothy J. Kamp, Lei Ye, Jianyi Zhang

**Affiliations:** ^1^ Department of Biomedical Engineering, School of Medicine and School of Engineering, University of Alabama at Birmingham, Birmingham, AL, United States; ^2^ Department of Medicine, School of Medicine and Public Health, University of Wisconsin-Madison, Madison, WI, United States; ^3^ Stem Cell and Regenerative Medicine Center, University of Wisconsin-Madison, Madison, WI, United States; ^4^ Department of Cell and Regenerative Biology, School of Medicine and Public Health, University of Wisconsin-Madison, Madison, WI, United States; ^5^ Department of Medicine/Cardiovascular Diseases, University of Alabama at Birmingham, Birmingham, AL, United States

**Keywords:** pluripotent stem cell, cardiomyocyte, suspension culture, maturation, organoids, biomanufacturing

## Abstract

Cardiomyocytes (CMs), endothelial cells (ECs), smooth-muscle cells (SMCs), and cardiac fibroblasts (CFs) differentiated from human induced-pluripotent stem cells (hiPSCs) are the fundamental components of cell-based regenerative myocardial therapy and can be used as *in-vitro* models for mechanistic studies and drug testing. However, newly differentiated hiPSC-CMs tend to more closely resemble fetal CMs than the mature CMs of adult hearts, and current techniques for improving CM maturation can be both complex and labor-intensive. Thus, the production of CMs for commercial and industrial applications will require more elementary methods for promoting CM maturity. CMs tend to develop a more mature phenotype when cultured as spheroids in a three-dimensional (3D) environment, rather than as two-dimensional monolayers, and the activity of ECs, SMCs, and CFs promote both CM maturation and electrical activity. Here, we introduce a simple and reproducible 3D-culture–based process for generating spheroids containing all four cardiac-cell types (i.e., cardiac spheroids) that is compatible with a wide range of applications and research equipment. Subsequent experiments demonstrated that the inclusion of vascular cells and CFs was associated with an increase in spheroid size, a decline in apoptosis, an improvement in sarcomere maturation and a change in CM bioenergetics.

## Introduction

The development of efficient protocols for differentiating human induced-pluripotent stem cells (hiPSCs) into cardiomyocytes (CMs), endothelial cells (ECs), smooth-muscle cells (SMCs), and cardiac fibroblasts (CFs) (Adams et al., 2013; Liu et al., 2016; Palpant et al., 2017; Zhu et al., 2017; Kwong et al., 2019; Zhang et al., 2019; Kahn-Krell et al., 2021) has led to the establishment of an array of utilizations in the field of cardiac regeneration. Although early proposed applications focused on direct injections (Menasché et al., 2001; Laflamme et al., 2007; Sanganalmath and Bolli, 2013; Ye et al., 2014) and the transplantation of engineered cardiac tissues (Schaefer et al., 2017; Shadrin et al., 2017; Gao et al., 2018) and sheets (Ishigami et al., 2018; Bayrak and Gümüşderelioğlu, 2019; Tsuruyama et al., 2019) to resupply cardiomyocytes, lost to injuries such as myocardial infarction (MI), the uses of these cardiac surrogates have expanded to include *in-vitro* modeling of myocardial disease (Long et al., 2018; Giacomelli et al., 2021), drug screening (Mathur et al., 2015; Huebsch et al., 2016; Mills et al., 2017), and exosome production (Liu et al., 2017). A wide range of formats has been explored to fulfill the needs of each potential application including patches (Schaefer et al., 2017; Shadrin et al., 2017; Gao et al., 2018), sheets (Ishigami et al., 2018; Bayrak and Gümüşderelioğlu, 2019; Tsuruyama et al., 2019), spheres (Fischer et al., 2018; Chang et al., 2020), wires (Jackman et al., 2016; Sun and Nunes, 2016), and decellularized tissues (Das et al., 2019; Alexanian et al., 2020; Hochman-Mendez et al., 2020). The utilization of these products and the needs of each use play a key role in the ideal option for each situation. However, there is a crucial need to establish tools for multiple applications related to MI and heart failure that can be easily reproduced and scaled (Zhang et al., 2021a).

Although current differentiation techniques can produce beating hiPSC-derived CMs in as little as 9 days (Lian et al., 2012; Zhang et al., 2012), the biomolecular (Cao et al., 2008; Synnergren et al., 2008; Xu et al., 2009; Synnergren et al., 2010), electrical (Caspi et al., 2009; Kim et al., 2010; Lee et al., 2011), and mechanical (Binah et al., 2007; Zhang et al., 2021b) properties of these newly differentiated cells tend to resemble those of fetal CMs, rather than the mature CMs of adult hearts (Robertson et al., 2013). Electrical stimulation (Chan et al., 2013; Richards et al., 2016; Ruan et al., 2016; LaBarge et al., 2019a) and mechanical stretching (Lux et al., 2016; Ruan et al., 2016; Zhang et al., 2017; LaBarge et al., 2019a) have been used to increase the maturity of engineered cardiac tissue but are difficult to apply until after the tissue is manufactured. Thus, the production of CMs for commercial and industrial applications will require more elementary methods for promoting CM maturation (Zhang et al., 2021a), such as manipulating the conditions of hiPSC-CM culture (Haishuang Lin et al., 2017; Parikh et al., 2017; Jackman et al., 2018; Selvaraj et al., 2019).

Another method to increase complexity and functionality of cardiac tissue models is to use additional cell types that more accurately recreate the myocardial environment. When cultured in a three-dimensional (3D) environment, rather than as two-dimensional monolayers, CMs coalesce into spheroids (Fischer et al., 2018; Chang et al., 2020) and tend to develop a more mature phenotype (Jha et al., 2016; Sacchetto et al., 2020; Kai-Li Wang et al., 2021). ECs and SMCs also promote CM maturation (Pinto et al., 2016; Ayoubi et al., 2017; Giacomelli et al., 2017; Zhu et al., 2017; Kwong et al., 2019) while facilitating oxygen and nutrient delivery, which improves cell survival (Garzoni et al., 2009; Zhang et al., 2021a; Pretorius et al., 2021), and CFs contribute to myocardial development both by producing components of the extracellular matrix (ECM) and by forming gap junctions with CMs to support electronic signal transduction (Zhang et al., 2019; Beauchamp et al., 2020; Giacomelli et al., 2020; Li et al., 2020; Pretorius et al., 2021). A range of ratios between these different cell types have been explored in previous studies but a relationship of CM:EC:SMC:CF of 4:2:1:1 is predominantly used (Gao et al., 2018; Arai et al., 2020; Beauchamp et al., 2020; Daly et al., 2021; Pretorius et al., 2021) as it roughly recapitulates the relationships found in native myocardium of myocytes predominating with endothelial cells compromising the greatest non-myocyte population (Banerjee et al., 2007; Pinto et al., 2016).

Along with the functionality and maturity requirements for broad cardiac tissue surrogate application two important biomanufacturing considerations are system format and scalability. A uniform shape, size, and culture condition that can function in a range of uses would allow for centralized production (Abbasalizadeh et al., 2017; Adil and Schaffer, 2017; Li et al., 2017; Tomov et al., 2019). Spherical spheroids cultured in suspension provide a format that is broadly compatible with existing research equipment, is highly movable, and can function as a building block for larger construct needs (Mattapally et al., 2018; LaBarge et al., 2019a; LaBarge et al., 2019b; Kim et al., 2020; Daly et al., 2021; Polonchuk et al., 2021). Additionally, using previously established CM spheroid production processes combined with whole spheroid fusion, a scalable spheroid biomanufacturing platform can be established that does not require dissociation (Beauchamp et al., 2015; Bin Lin et al., 2017; Miwa et al., 2020; Kahn-Krell et al., 2021).

For the experiments described in this report, we differentiated hiPSCs into CMs, ECs, SMCs, and CFs and then combined the differentiated cells in a 3D culture environment, where they formed spheroids containing all four cardiac-cell types (i.e., cardiac microtissues). Subsequent analyses suggested that the inclusion of vascular cells and CFs increased spheroid size, reduced cellular apoptosis, and tended to promote sarcomere maturation and CM energy production. This process, with the potential to be scaled, advances on previous cardiac spheroid models (Giacomelli et al., 2017; Voges et al., 2017; Lee et al., 2019b; Helms et al., 2019; Keung et al., 2019; Beauchamp et al., 2020; Buono et al., 2020; Giacomelli et al., 2020; Israeli et al., 2020; Kupfer et al., 2020; Thomas et al., 2021) by producing large diameter tissues with cells from a single iPSC line, no exogenous matrix, and a reproducible product.

## Materials and methods

### hiPSC culture and expansion

All cells used in the study were differentiated from human induced pluripotent stem cell (hiPSC) line LZ-hiPSC5 which was reprogramed from human cardiac fibroblasts as described previously (Zhang et al., 2014). hiPSCs were cultured on 6-well plates in mTeSR Plus (STEMCELL Technologies) for 3 days, detached with gentle cell dissociation reagent (GCDR) (STEMCELL Technologies), resuspended in 40 ml TeSR E8 3D seed media (STEMCELL Technologies) supplemented with 10 µM Y27632 at a density of 1.5 × 10^5^ cells/mL, and then cultured in a 125 ml Erlenmeyer flask on a Belly Dancer orbital shaker (IBI Scientific) at a speed of 4.75. On each of the following 2 days, 1.2 ml of feed medium was added to the flask, and after an additional day, half of the culture volume was replaced with fresh seed medium. After 4 days of culture on the orbital shaker, aggregates were dissociated with GCDR for 8 min, broken into smaller cell clumps via pipetting through a 37-µm reversible strainer (STEMCELL Technologies), and then diluted to a density of 1.5 × 10^5^ cells/mL. Cells were cultured as previously described (Kahn-Krell et al., 2021) for four more days before differentiation was initiated.

### CM differentiation

Cardiomyocyte (CM) differentiation was performed as described previously (Kahn-Krell et al., 2021). Briefly, differentiation was initiated on differentiation day (dD) 0 by replacing the culture media with RPMI 1640 supplemented with 1 × B27 without insulin (RPMI/B27–), 6 µM CHIR99021, and 10 µM Y-27632 at a density of 1.5 × 10^6^ cells/mL. On dD1, the media was replaced with a 1.2-fold volume of RPMI/B27–, 1 µM CHIR99021, and 10 µM Y-27632, and the cells were cultured for two more days. On dD3, 70% of the culture media was replaced with RPMI/B27– supplemented with 10 µM IWR-1-endo; on dD5, the media was changed to RPMI/B27–; and on dD7, the media was changed to RPMI1640 supplemented with B27 with insulin (RPMI/B27+) in a volume equivalent to the volume used on dD0. Metabolic purification of the differentiated CMs was initiated on dD9 by changing the media to RPMI1640 without glucose supplemented with B27+ and 0.12% (w/w) sodium DL-lactate (Millipore Sigma). Three days later (on dD12), the purification media was replaced with RPMI/B27+, and the medium was refreshed every 5 days until spheroid assembly. Spheroid assembly was performed no more than 30 days after differentiation was initiated and as close to dD12 as possible.

### EC differentiation

Endothelial-cell (EC) differentiation of hiPSCs was performed in monolayers of cultured hiPSCs with the STEMdiff Endothelial Differentiation Kit (STEMCELL Technologies) as directed by the manufacturer’s instructions. Briefly, hiPSCs were seeded into mTeSR Plus (STEMCELL Technologies) in a 6 well plate at a density of 5.0 × 10^4^ per well and then on dD1 and dD2, the medium was changed to 3 ml STEMdiff Mesoderm Induction Medium (STEMCELL Technologies). The medium was replaced with 4 ml of STEMdiff Endothelial Induction Medium (STEMCELL Technologies) on dD3 and refreshed on dD5. On dD7, the cells were dissociated with ACCUTASE (Corning), transferred into a fibronectin-coated T75 flask, and cultured in EGM-2 MV (Lonza) supplemented with SB431542 (Fischer Scientific) until 100% confluent. Purification was performed by dissociating the cells, resuspending them in cold Dulbecco phosphate-buffered saline (DPBS) with 2% fetal bovine serum (FBS) at a density of 1 × 10^6^ cells/100 μL, and then collecting cells that expressed both CD31 and CD144 via flow cytometry on a BD FACS Aria II instrument; 100-µL samples were labeled by incubating them with 5 µL of AlexaFluor-conjugated CD31 and 20 µL of phycoerythrin-conjugated CD144 antibodies for 45 min on ice and then washed in DPBS.

### SMC differentiation

hiPSCs were treated via the EC differentiation protocol through dD7 and then differentiated into smooth-muscle cells (SMCs) as described previously (Yang et al., 2016). Briefly, the cells were cultured until 80% confluent, and then the media was changed to high-glucose DMEM supplemented with 5% FBS, 5 ng/ml platelet-derived growth factor beta (PDGF-β), and 2.5 ng/ml transforming growth factor beta (TGFβ). The media was changed every 3 days for 9 days and then replaced with SmGM-2.

### Cardiac fibroblast differentiation

When hiPSCs reached 100% confluency the medium was changed to RPMI/B27–insulin supplemented with 12 μM CHIR99021 (Tocris) for 24 h, RPMI/B27–insulin for 24 h, and then cardiac fibroblast differentiation basal (CFBM) medium (DMEM, high glucose with HAS, linoleic acid, lecithin, ascorbic acid, GlutaMAX, hydrocortisone hemisuccinate, rh insulin) supplemented with 75 ng/ml bFGF (WiCell Research Institute) for 18 days; the CFBM/bFGF medium was changed every other day.

### CM, EC, SMC, and CF purity assessments

Cells were dissociated, washed in DPBS, fixed with 4% paraformaldehyde (PFA) for 15 min, washed three times, permeabilized with 0.1% Triton-X in DPBS, and blocked with 4% bovine serum albumin (BSA) and 4% FBS in DPBS for 30 min each; then, the cells were incubated with lineage-specific antibodies (Supplemental Table S1) for 1 h and analyzed on an Attune NxT flow cytometer (Thermo Fisher Scientific).

### Spheroid fabrication and culture

A 2 ml sample of CM spheroids were treated with CM dissociation media (CMDM) (STEMCELL Technologies) for 15 min at 37°C with periodic mixing then counted to determine culture density. ECs, SMCs, and CFs were dissociated from monolayer cultures and 5.0 × 10^6^, 2.5 × 10^6^, and 2.5 × 10^6^ of each respectively were combined and resuspended in 1 ml of spheroid media (OM) (Supplemental Table S3). Based on cell counts 1.0 × 10^7^ total CMs were collected as whole spheroids in a 50 ml conical tube and the media was replaced with 19 ml OM. After addition of the other cell types the contents were mixed well and 200 µL was transferred to each well of a 96-Well, Nunclon Sphera-Treated, U-Shaped-Bottom Microplate (Thermo Fisher Scientific) to produce 96 spheroids. The media was refreshed every 2 days for 7 days, and then the spheroids were transferred to 6-well Ultra-Low Attachment Microplates (Corning), with no more than 16 spheroids per well. Plates were maintained on a belly dancer shaker (IBI Scientific) at speed of 4.75, and the media was exchanged every 4–7 days.

### Spheroid size measurements

Spheroids were photographed with an Olympus CKX53 microscope at ×4 magnification, and spheroid diameters were determined with a modified ImageJ macro (Ivanov et al., 2014) as indicated in the Supplemental Information.

### Quantitative polymerase chain reaction (qPCR)

RNA was extracted with TRIZOL (Thermo Fisher) and purified on Direct-zol RNA Miniprep Plus (Zymo Research) columns as directed by the manufacturer’s protocol. Reverse transcription was performed with Superscript IV VILO Master Mix (Invitrogen), and samples were diluted to 5 ng/μL. Each qPCR reaction was performed with 5 ng of cDNA, 0.5 µM primers (Supplemental Table S2), and PowerUp SYBR Green Master Mix (Applied Biosystems), and analysis was conducted on a QuantStudio Real-Time PCR Machine (Applied Biosystems). Results for each CT value were normalized to intrinsic glyceraldehyde phosphate dehydrogenase (GAPDH) abundance and to the CT value determined on the first day after spheroid assembly for each batch of spheroids. Data was collected from four independent batches of spheroids for each gene.

### Western blotting

Spheroids were digested in 100 µL RIPA Buffer (Sigma-Aldrich); then, protein lysates were collected, mixed with ×1 Halt proteinase inhibitor cocktail mix (Thermo Scientific), and sonicated twice in 3-s intervals at 50% power. Sample concentrations were determined via Pierce BCA Protein Assay (Thermo Scientific), and then samples containing ×4 Laemmli Sample Buffer (Bio-Rad), 2 Mercaptoethanol (Bio-Rad), and 7.5 µg protein were run on an Expressplus PAGE 4–20% Gel (GenScript) at 200 V for 30 min. Proteins were transferred to nitrocellulose membranes with a Trans-Blot Turbo Transfer System (Bio-Rad); then, the membranes were blocked in 5% Blotting Grade Blocker Non Fat Dry Milk (Bio-Rad) for 30 min and stained with primary and secondary antibodies (Supplemental Table S1) for 1 h each. Blots were incubated for 5 min in Pierce SuperSignal West Pico PLUS Chemiluminescent Substrate (Thermo Scientific) and imaged with the ChemiDoc Touch Imaging System (Biorad). Protein bands were quantified with Image Lab Software (Bio-Rad); results for each sample were normalized to intrinsic beta actin abundance and to measurements made on the first day after spheroid assembly for each batch of spheroids. Data was collected from four independent batches of spheroids for each protein.

### Spheroid preservation and sectioning

Spheroids were fixed in 4% formaldehyde (Pierce) for 15 min, placed in 30% sucrose at 4 °C overnight, and then then embedded in Tissue-Plus O.C.T Compound (Fisher Scientific) for histological analysis. Blocks were cut into 10-µm sections, mounted on charged glass microscope slides (Globe Scientific) and then stored at −20°C.

### Masson-trichrome staining

Slides containing sections obtained at 100-µm intervals (i.e., every 10th section) were fixed in Bouin’s Solution at 55°C for 1 h and then treated with Weigerts iron hematoxylin working solution for 10 min, with Biebrich scarlet-acid fuchsin for 5 min, with phosphomolybdic acid-phosphotungstic acid for 5 min, with aniline blue solution for 5 min, and with 1% acetic acid for 1 min; then, the sections were dehydrated in 95% alcohol for 2 min, cleared with 2 changes of xylene, and mounted with permount and coverslips overnight. Sections were imaged with a ×10 objective on an Olympus B×51 Fluorescence Microscope; when multiple fields of view were required for a single section, the images were stitched together with Photoshop software.

### Apoptosis

The spheroid core was identified by determining which Masson-trichrome–stained sections had the greatest surface area; then, sections from the core were stained using the *In Situ* Cell Death Detection Kit, TMR red (Sigma) according to the manufacturers protocol. Briefly, sections were fixed in 4% PFA for 15 min, permeabilized in 0.1% Triton X-100 and 0.1% sodium citrate for 2 min on ice, incubated with TUNEL reaction mixture and 4′, 6-diamidino-2-phenylindole (DAPI) for 1 h at 37°C, mounted in VECTASHIELD hardset Antifade Mounting Medium, and visualized via confocal laser scanning (Olympus FV3000 confocal microscope). Sections were evaluated for at least two spheroids in each of four batches, and positively stained cells or the size of stained regions were quantified with a modified ImageJ macro (Ivanov et al., 2014) as indicated in the Supplemental Information.

### Immunostaining and apoptosis detection

The spheroid core was identified by determining which Masson-trichrome–stained sections had the greatest surface area; then, sections from the core were fixed in 4% PFA for 15 min, blocked, permeabilized in 10% donkey serum, 10% Tween20, 3% BSA, and 0.05% Triton-X for 30 min, incubated with primary antibodies (Supplemental Table S1) at room temperature for 45 min, washed with PBS (3 washes, 5 min per wash), incubated with DAPI and fluorescent secondary antibodies at room temperature for 45 min, mounted in VECTASHIELD hardset Antifade Mounting Medium, and visualized via confocal laser scanning (Olympus FV3000 confocal microscope). Sections were evaluated for at least two spheroids in each of four batches, and positively stained cells or the size of stained regions were quantified with a modified ImageJ macro (Ivanov et al., 2014) as indicated in the Supplemental Information.

### ATP, NAD, and NADH quantification

ATP content was measured using a luminescent ATP detection Assay kit (ab113849; Abcam) following the manufacturer’s protocol. The amount of ATP was normalized to the total protein content, which was determined using a protein assay (23227, ThermoFisher). The data was presented as nanomoles per milligram protein. Total NAD+ and NADH levels were measured by colorimetric kit (ab65348; Abcam) according to manufacturer’s instruction. cAMP level was measured by Direct cAMP Enzyme-linked Immunosorbent Assay (ELISA) using a direct cAMP ELISA kit (ADI-900-066A, Enzo Life Sciences) following the manufacturer’s instructions. Tissue cAMP level was normalized to the total protein content and presented as picomole per milligram protein.

### Microelectrode array (MEA)

CytoView MEA 24 well plates (Axion Biosystems) were precoated with 3 μg/ml fibronectin and incubated at 37°C for 1 h. Whole spheroids (1 per well) or CMs collected from dissociated spheroids were added to each well of the MEA plate. Isolated CMs were obtained by treating spheroids with CMDM and periodic pipetting for 30 min at 37°C, selected with the EasySep Human PSC-Derived Cardiomyocyte Enrichment Kit (StemCell), and resuspended at a concentration of 1.2 × 10^7^ cells/mL in CM support media (CMSM) (StemCell Technologies); 5 µL of resuspended CMs were added to each well. Whole spheroids were analyzed 24–48 h after plating, and CMs were analyzed 7 days after plating. Field potential and contractility measurements were collected on a Maestro Edge apparatus (Axion Biosystems) and analyzed by using the Cardiac Module in Axion Navigator software. Action potential durations (APDs) were determined using the LEAP assay and characterized using the Cardiac Analysis Tool.

### Transmission electron microscopy (TEM)

Spheroids were transferred onto a fibronectin-coated (1 μg/ml), 0.4-µm pore Transwell Polycarbonate Membrane and cultured for 3 days; then, the membranes were fixed in 2.5% glutaraldehyde solution for 1 h at 4°C and delivered to the UAB High-Resolution Imaging Facility. Sample blocks were sectioned along the width of the transwells with a diamond knife, and samples were mounted and viewed with a Tecnai Spirit T12 Transmission Electron Microscope. At least 4 images were collected for each spheroid, and sarcomere lengths and widths were determined for all sarcomeres in an image by using the line-measure tool in ImageJ software. Sarcomere lengths were determined as the distance from z-line to z-line and widths were determined as the distance from one end to the other of a continuous z-line.

### Statistical analysis

Data are presented as mean ± SEM, as box-and-whisker plots, or as violin plots, and significance was evaluated via the Student’s *t*-test or analysis of variance (ANOVA). Analyses were performed with GraphPad Prism8 software (GraphPad Prism, RRID:SCR_002798), and *p* < 0.05 was considered significant.

## Results

### The inclusion of hiPSC-ECs, -SMCs, and CFs in CM spheroids increased spheroid size and improved cell viability

**FIGURE 1:**
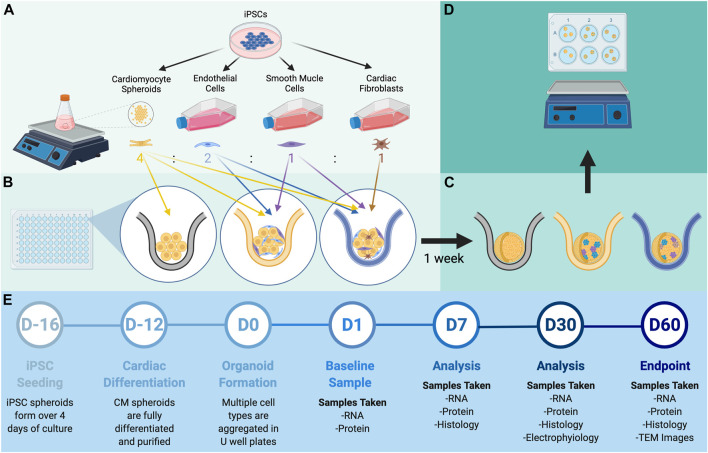
Experimental design. **(A)** hiPSCs were differentiated into CMs, ECs, SMCs, and CFs via established methods. **(B)** Spheroids containing all four cell types (C4 spheroids) were produced by combining a 4:2:1:1 ratio of CMs, ECs, SMCs, and CFs in 96-well U plates and allowing the cells to fuse for 7 days; then, **(C)** the spheroids were transferred to 6-well plates and **(D)** cultured with shaking for to 60 days. **(E)** Procedures for spheroid manufacture and experimental analyses are displayed on a timeline.

**FIGURE 2:**
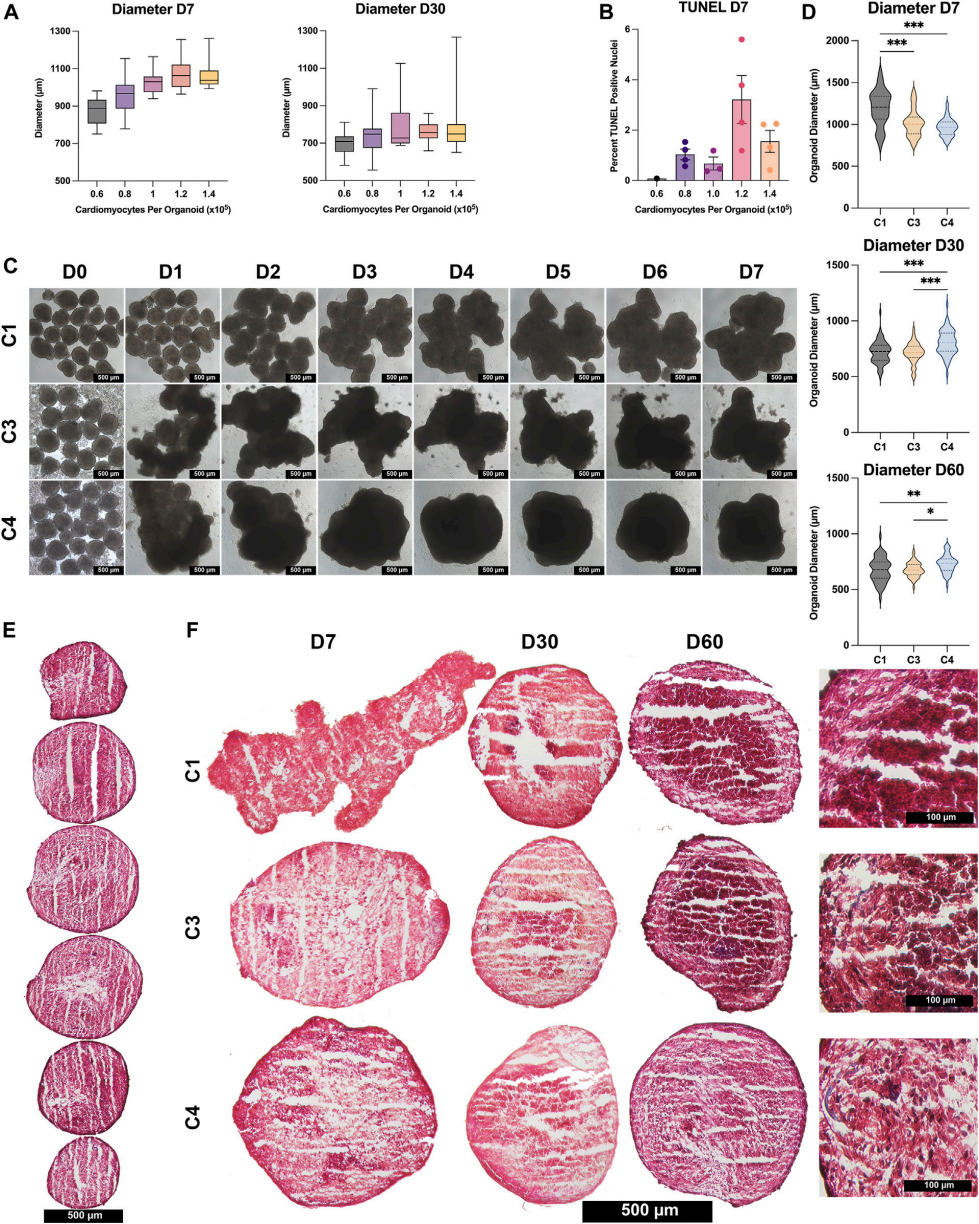
Size and histological assessments. **(A)** C4 spheroids were generated from the indicated CM population sizes (0.6 × 10^5^ black; 0.8 × 10^5^ purple; 1.0 × 10^5^ pink; , 1.2 × 10^5^ orange; , 1.4 × 10^5^ yellow), and spheroid diameters were measured on D7 and D30; results were displayed as box-whisker plots. **(B)** C4 spheroids were cut into 10-μm sections, and the sections were stained via terminal deoxynucleotidyl transferase dUTP nick end labeling (TUNEL); then, apoptosis was quantified as the percentage of TUNEL-positive cells in 4 spheroids/group. **(C)** Images of C1, C3, and C4 spheroids generated from an initial population of 1 × 10^5^ CMs were obtained each day from D0–D7 (bar = 500 μM). **(D)** Spheroid diameters of all 3 groups (C1 black; C3 yellow; C4 blue) were measured on D7, D30, and D60; results are displayed as violin plots. **(E)** Spheroids were cut into 10-µm sections and stained with Masson-trichrome; representative images are displayed for every 10th sequential section from a C4 spheroid on D60 (bar = 500 μM). **(F)** Representative images are displayed for the centermost sections from C1, C3, and C4 spheroids at the indicated time points (bar = 500 μM for the first three columns, and bar = 100 μM for the far-right column); muscle fibers appear red and collagen fibers appear blue. (*p < 0.05, **p < 0.01, ***p < 0.001; n > 30 per group; *p*-values determined as mean ± SEM).

hiPSCs were differentiated into CMs, ECs, SMCs, and CFs via published protocols or the use of commercially available kits (Yang et al., 2016; Zhang et al., 2019; Kahn-Krell et al., 2021), and flow-cytometry analyses with lineage-specific antibodies [CMs: cardiac troponin T (cTnT), ECs: CD144 and CD31, SMCs: smooth-muscle actin (SMA), CFs: TE-7] confirmed that the purity of each differentiated cell population exceeded 95% (Supplementary Figures S1–S4). Spheroids containing CMs alone (C1) and spheroids containing a 4:2:1 ratio of CMs, ECs, and SMCs (C3) or a 4:2:1:1 ratio of CMs, ECs, SMCs, and CFs (C4) were produced by culturing the indicated proportions of cell types in low-attachment 96-well U plates for 1 week to promote aggregation and fusion (Figures 1A,B), and then in low-attachment 6-well plates on an orbital shaker for the remainder of the culture period (Figures 1C,D). D0 was defined as the day the spheroids/spheroids were initially assembled, and samples were obtained after one (D1), seven (D7), 14 (D14), 30 (D30), and 60 (D60) days of culture for characterization (Figure 1E); synchronized beating was observed in all constructs throughout the culture period. Size optimization studies determined that the mean diameter of C4 spheroids on D30 was larger (though not significantly) when the spheroids were generated from $1.0\times 10^{5}$ seeded CMs (∼801 µm) than from other initial CM population sizes (698–791 µm) (Figure 2A), with no significant increase in the proportion of apoptotic cells (Figure 2B). Thus, all spheroids and spheroids produced for subsequent experiments were constructed with 1.0 × 10^5^ CMs.

**FIGURE 3:**
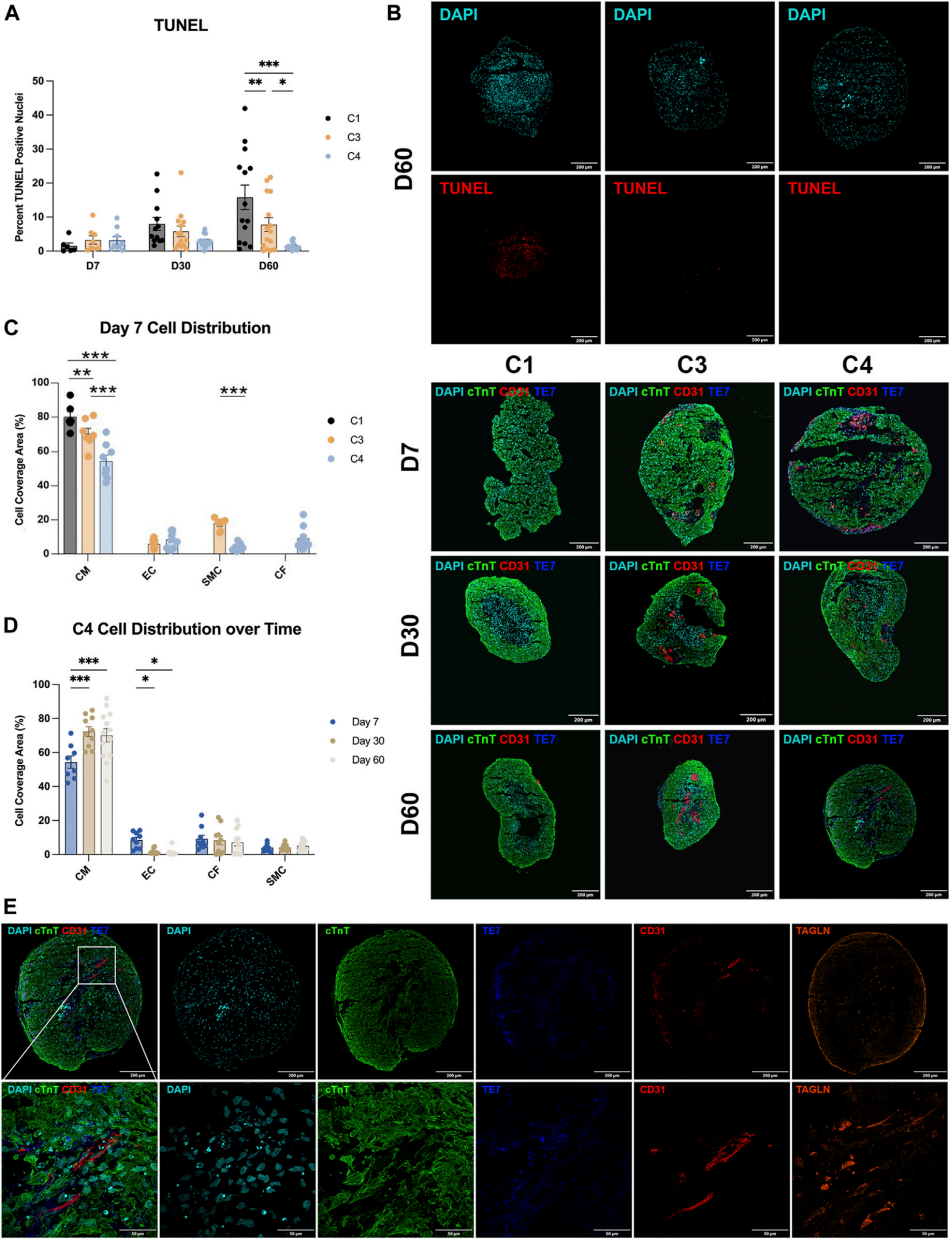
Apoptosis and cell-occupancy measurements. **(A)** C1, C3, and C4 spheroids were collected at the indicated time points and cut into 10-µm sections; then, the surface areas of every 10th section were measured, and the centermost section (i.e., the one with the largest surface area) was TUNEL-stained. Nuclei were counterstained with DAPI, and apoptosis was quantified as the percentage of TUNEL-positive cells. Representative images are displayed for the centermost sections from each group on D60. **(B)** Centermost sections were stained with cTnT, CD31, and TE-7 antibodies to visualize CMs, ECs, and CFs, respectively, and nuclei were counterstained with DAPI; representative images are displayed for each group on D7, D30, and D60. **(C,D)** CM, EC, and CF occupancy were quantified as the percentage of total surface area that was positive for expression of the corresponding marker protein and summarized **(C)** for C1, C3, and C4 on D7 and **(D)** for C4 on D7, D30, and D60. **(E)** Representative sections from a C4 spheroid on D60 are displayed at high magnification to demonstrate the presence of elongated ECs. (*p < 0.05, **p < 0.01, ***p < 0.001; n > 6 per group).

C4 spheroids coalesced into a largely spherical shape within 3–4 days of seeding, while the architecture of the C3 spheroids and (especially) C1 spheroids remained irregular through at least D7 (Figure 2C). C4 spheroid diameters were also significantly smaller on D7 (C1: 1,198 ± 38.2 µm; C3: 1,016 ± 21.1 µm; C4: 964 ± 12.6 µm, *p* < 0.001 versus C1 and C3), but the trend was reversed on D30 (C1: 719 ± 11.9 µm; C3: 714 ± 10.4 µm; C4: 804 ± 13.9 µm, *p* < 0.001 versus C1 and C3) and D60 (C1: 676 ± 14.7 µm; C3: 678 ± 8.8 µm; C4: 730 ± 11.7 µm, *p* < 0.01 versus C1, *p* < 0.05 versus C3), when C4 spheroids were significantly larger than the other two constructs (Figure 2D). These differences may be accounted for by proliferation of non-myocytes in the C3 and C4 groups however, over time all groups showed trends of decreasing diameter and further immunostaining (Figure 3) did not show significant increases in ECs, SMCs, or CFs. Observations in Masson-trichrome stained sequential sections (Figure 2E) suggested that the density of muscle cells and fibers increased from D7 to D60 in all constructs, but only C3 and C4 spheroids displayed evidence of collagen formation (Figure 2F), which is consistent with the absence of ECM-producing ECs, SMCs, or CFs (Lee et al., 2019a; Strikoudis et al., 2019) in C1 spheroids. Furthermore, although assessments of apoptosis (TUNEL staining) did not differ significantly among groups on D7, apoptotic cells became increasingly common in C1 spheroids and (to a lesser extent) C3 spheroids during the culture period, while the proportion of apoptotic cells in C4 spheroids remained stable through at least D60, when apoptotic cells were significantly less common in C4 spheroids than in the other two constructs and in C3 spheroids than in C1 spheroids (Figure 3A). Apoptotic cells also tended to be located toward the center of C1 spheroids, which suggests that the vascular cells and CFs present in C4 spheroids facilitated access of the culture medium to the spheroid interior. These findings are consistent with previous work supporting the crucial role that non-myocytes play in promoting CM survival particularly where nutrient and oxygen diffusion is limited through release of paracrine factors (Ye et al., 2014; Hodgkinson et al., 2016; Giacomelli et al., 2017; Giacomelli et al., 2020; Munarin et al., 2020; Pretorius et al., 2021).

### hiPSC-CMs occupied a progressively larger proportion of C4 spheroids over time

The distribution of cell types during the culture period was evaluated in sections stained for the expression of cTnT, CD31, and the fibroblast marker TE-7 (Figure 3B), as well as with SMC marker transgelin (TAGLIN). On D7, the proportion of cTnT-positive surface area (i.e., CM occupancy) was significantly lower in C4 spheroids than in the other two constructs, and in C3 spheroids than in C1 spheroids (Figure 3C), which is consistent with the initial composition of cell types used during spheroid construction. In C4 spheroids, CM occupancy increased from D7 to D30, while EC occupancy declined over the same period (Figure 3D), perhaps because the ECs grew from centrally located clumps on D7 into elongated structures that permeated the entire spheroid on D30 and D60 (Figure 3E). CM and EC occupancy remained largely stable in C4 spheroids from D30 to D60, while SMC and CF occupancy did not change significantly throughout the 60-day culture period. Although some restructuring occurred, immunofluorescent imaging studies illustrated sparse and non-uniform distribution of non-myocytes throughout the time course suggesting that even small amounts of these cells can provide the necessary benefit to CM survival. Low levels of CD31 expression were also observed in C1 spheroids, and both C3 spheroids and C1 spheroids contained a very small number of TE-7–positive cells, perhaps because growth factors present in the culture medium (e.g., VEGF, FGF) induced EC- and CF-like phenotypes in hiPSC-derived cells that were not fully differentiated before the constructs were assembled.

### hiPSC-CMs in C4 spheroids matured during the culture period

**FIGURE 4:**
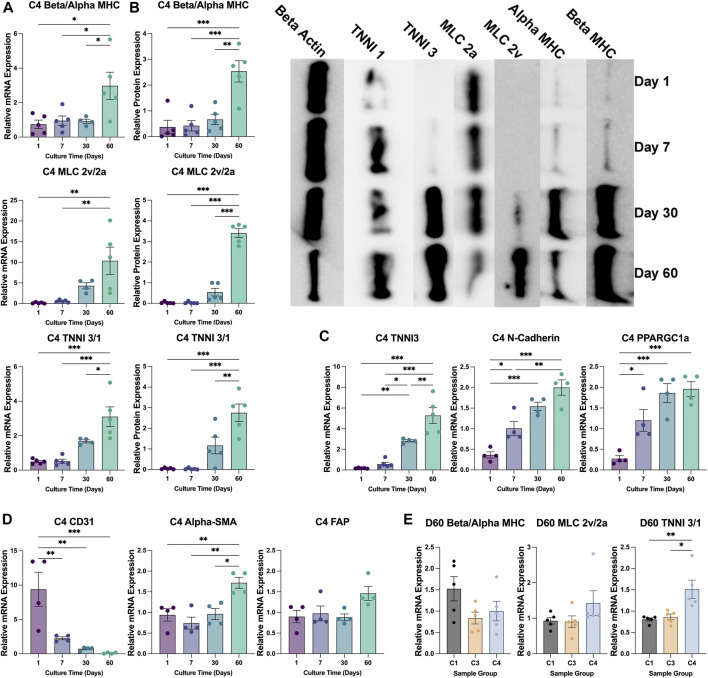
Changes in patterns of gene expression during culture. **(A,B)** The abundance of beta MHC, alpha MHC, MLC 2v, MLC 2a, TNNI3, and TNNI1 **(A)** mRNA and **(B)** protein were evaluated *via* qPCR and Western blot, respectively, in C4 spheroids at the indicated time points (D1 purple; D7 blue; D30 teal; D60 green); then, the ratios of expression for the MHC (top), MLC (middle), and TNNI isoforms (bottom) were calculated. A representative Western blot of protein expression is displayed on the right of panel **(B) (C,D)** The abundance of **(C)** TNNI3, N-cadherin, and PPARGC1a mRNA and of **(D)** CD31, alpha-SMA, and FAP mRNA was measured via qPCR in C4 spheroids at the indicated time points. **(E)** The ratios of mRNA abundance for the MHC, MLC, and TNNI isoforms was calculated for C1, C3, and C4 spheroids on D60 (C1 black; C3 yellow; C4 blue). mRNA measurements were normalized to intrinsic GAPDH mRNA abundance, and protein measurements were normalized to intrinsic beta actin abundance. (*p < 0.05, **p < 0.01; n > 4 per group).

CM maturation and ventricular specification is associated with increases in the ratios of expression for beta versus alpha myosin heavy chain (beta:alpha MHC), ventricular versus atrial myosin light-chain 2 (MLC 2v:2a), and cardiac (type 3) versus slow-skeletal (type 1) troponin I (TNNI 3:1) (Guo and Pu, 2020; Karbassi et al., 2020). When calculated from measurements of either mRNA (Figure 4A) or protein (Figure 4B) abundance, each of the three ratios increased significantly in C4 spheroids throughout the culture period, including from D30 to D60 for all except the ratio of MLC 2v:2a mRNA, and the abundance of structural (TNNI3), electromechanical (N-cadherin), and metabolic [peroxisome proliferator-activated receptor gamma coactivator 1-alpha (PPARGC1a)] markers for CM maturation significantly increased through at least D30 (Figure 4C). Alpha-SMA expression also increased significantly throughout the culture period, while CD31 expression significantly declined, and the expression of fibroblast activating protein (FAP) was largely unchanged (Figure 4D). Collectively, these observations indicate that the maturation and modification of both CMs and vascular cells continues for at least 2 months in cultured C4 spheroids.

### The inclusion of hiPSC-ECs, -SMCs, and CFs increased sarcomere maturation and CM energy production in cardiac spheroids

**FIGURE 5:**
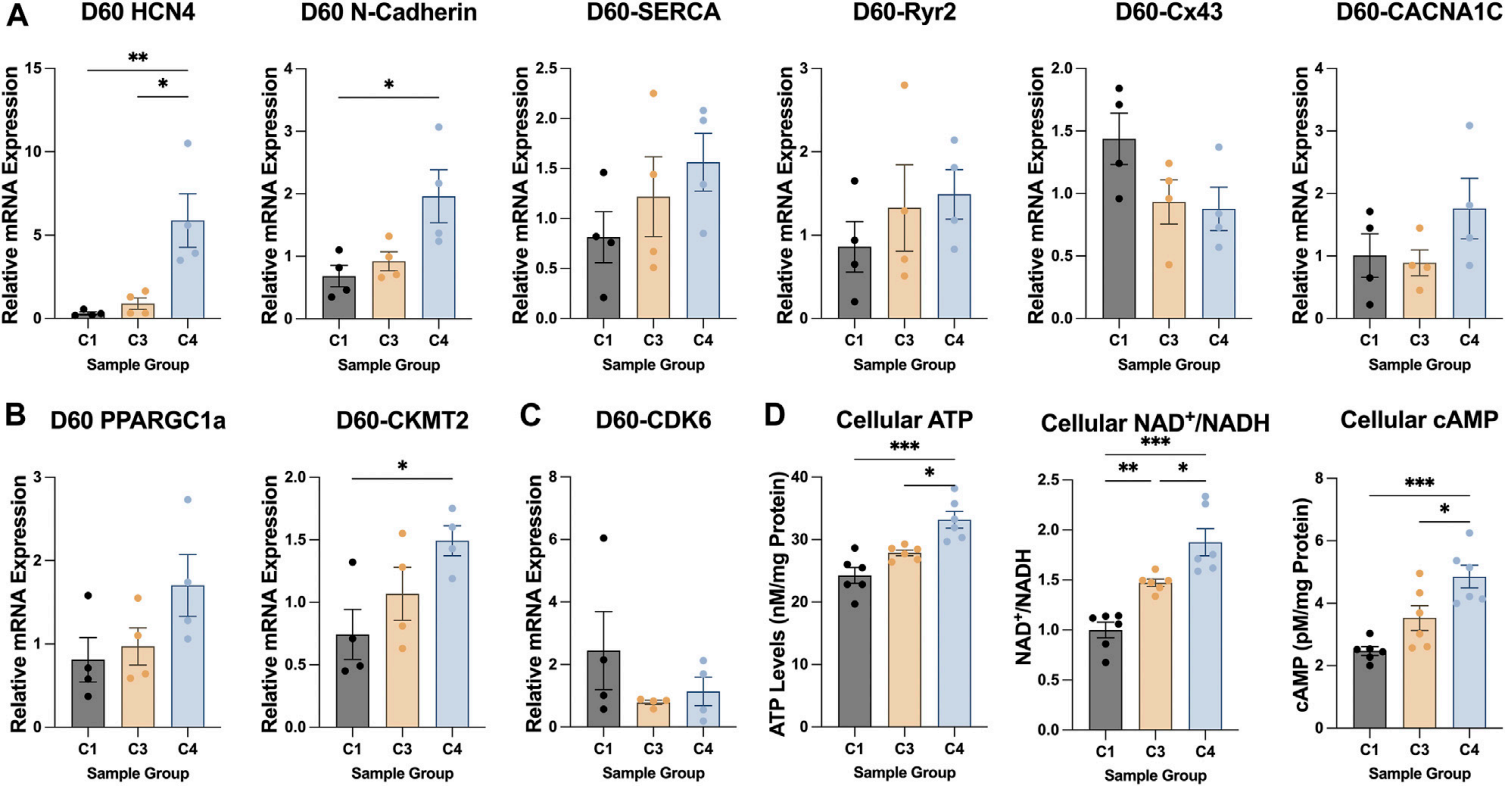
Patterns of metabolic, electrical, and cell-cycle gene expression in C1, C3, and C4 spheroids. The magnitude of expression for genes that contribute to CM **(A)** electrical conduction (HCN4, N-cadherin, SERCA, Ryr2, Cx43, CACNA1C), **(B)** metabolism (PPARGC1a, CKMT2), and **(C)** cell-cycle activity (CDK6) was evaluated in C1, C3, and C4 spheroids on D60 (C1 black; C3 yellow; C4 blue) via qPCR; measurements were normalized to intrinsic GAPDH mRNA abundance. **(D)** ATP abundance, the NAD/NADH ratio, and cellular cAMP was evaluated in C1, C3, and C4 spheroids on D60 (C1 black; C3 yellow; C4 blue) *via* luminescent ATP detection assay, colorimetric assay, and direct cAMP ELISA respectively (*p < 0.05, **p < 0.01; n > 4 per group).

**FIGURE 6:**
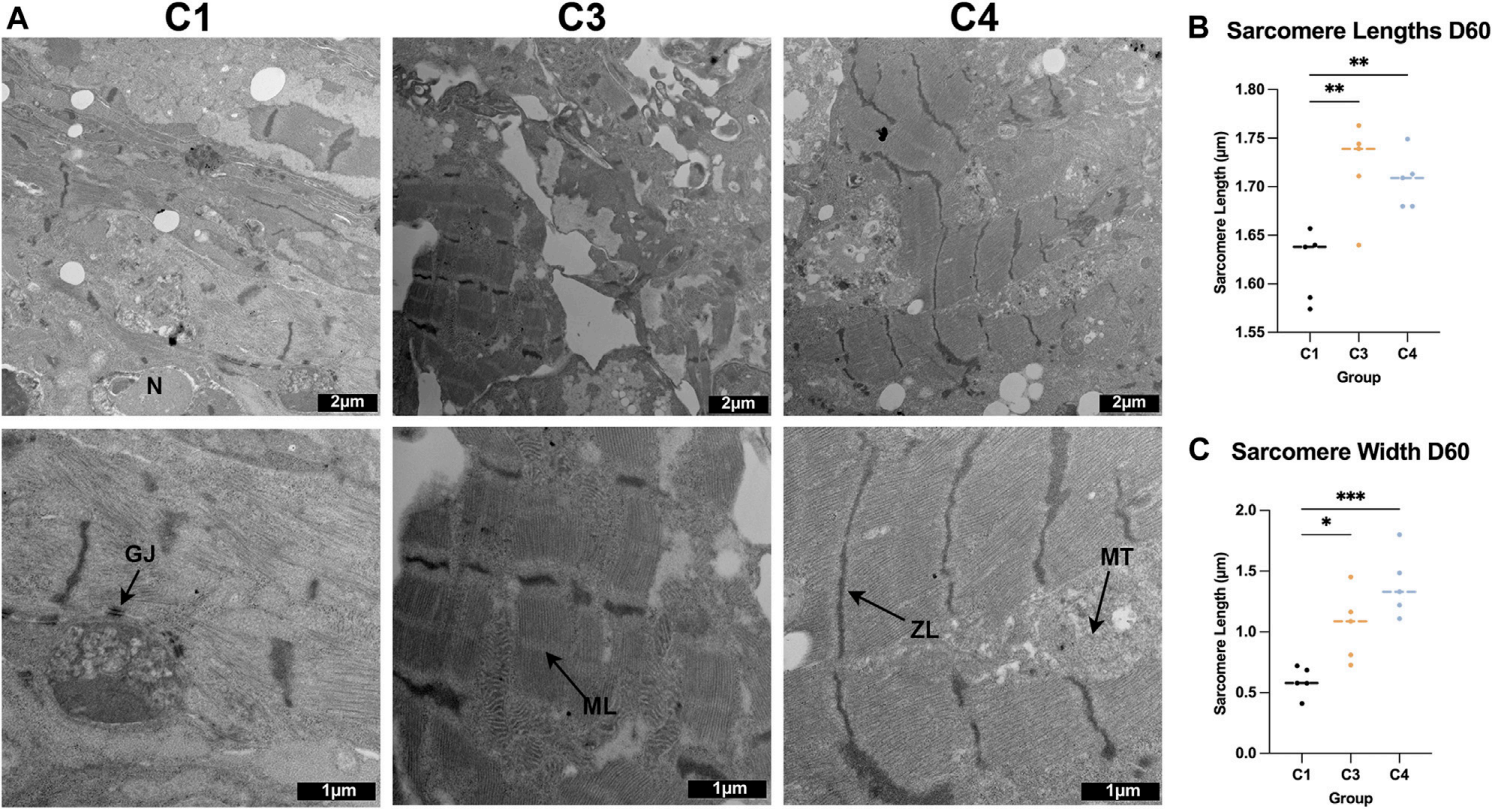
TEM assessments of the ultrastructure of C1, C3, and C4 spheroids. **(A)** Whole C1, C3, and C4 spheroids on day 60 were sectioned and imaged via TEM (bar = 2 μM for the top row, and bar = 1 μM for the bottom row); ZL: Z-line, ML: M-line, MT: mitochondria, GJ: gap junction, N: nucleus. **(B)** Sarcomere lengths were calculated by measuring the distance between adjacent Z-lines. **(C)** Sarcomere widths were measured as the length of each uninterrupted Z-line. (*p < 0.05, **p < 0.01, ***p < 0.001; n > 5 spheroids per group).

The TNNI 3:1 mRNA ratio was also significantly higher in C4 spheroids than in C3 spheroids or C1 spheroids on D60, but the ratios of beta:alpha MHC and MLC 2v:2a mRNA did not differ significantly among the three groups (Figure 4E). mRNA levels on D60 for a panel of genes that participate in CM electrical conduction [hyperpolarization activated cyclic nucleotide gated potassium channel 4 (HCN4), N-cadherin, sarcoplasmic/endoplasmic reticulum calcium ATPase (SERCA), ryanodine receptor 2 (Ryr2), connexin 43 (Cx43), calcium voltage-gated channel subunit alpha 1C (CACNA1C); Figure 5A] and metabolism [PPARGC1a, creatine kinase, mitochondrial 2 (CKMT2); Figure 5B] also tended to be more highly expressed in C4 spheroids; however, the only differences that reached statistical significance were for CKMT2 and N-cadherin, which were significantly greater in C4 spheroids than in C1 spheroids, and for HCN4, which was significantly greater in C4 spheroids than in either of the other two groups. These expression level differences observed on day 60 were found to be mostly absent when the same genes we examined on day 7 after formation (Supplementary Figure S5). Notably, mRNA levels for both Cx43 and the cell-cycle regulatory molecule cyclin-dependent kinase 6 (CDK6) (Figure 5C) appeared to be lower in C4 spheroids than in C1 spheroids, but not significantly, while ATP levels, the ratio of NAD:NADH, and cAMP levels were significantly greater in C4 spheroids than in C1 spheroids (Figure 5D). Furthermore, observations in transmission electron microscopy (TEM) images collected on D60 confirmed that all three spheroid constructs contained Z-lines and gap junctions, but more complex structures, such as M-lines, I-bands, and A-bands, were observed only in C3 and C4 spheroids. This was notably a qualitative improvement from images collected on spheroids at D30 (Supplementary Figure S6). Contractile fibers also appeared to be more organized, and mitochondria better aligned, in C4 spheroids (Figure 6A), and both sarcomere lengths (Figure 6B) and widths (Figure 6C) were significantly greater in C4 spheroids than C1 spheroids. Thus, the inclusion of ECs, SMCs, and CFs in cardiac spheroids tended to promote sarcomere maturation and CM energy production.

### Field-potential duration and conduction velocity were greater in CMs from C4 spheroids than from C1 spheroids

**FIGURE 7:**
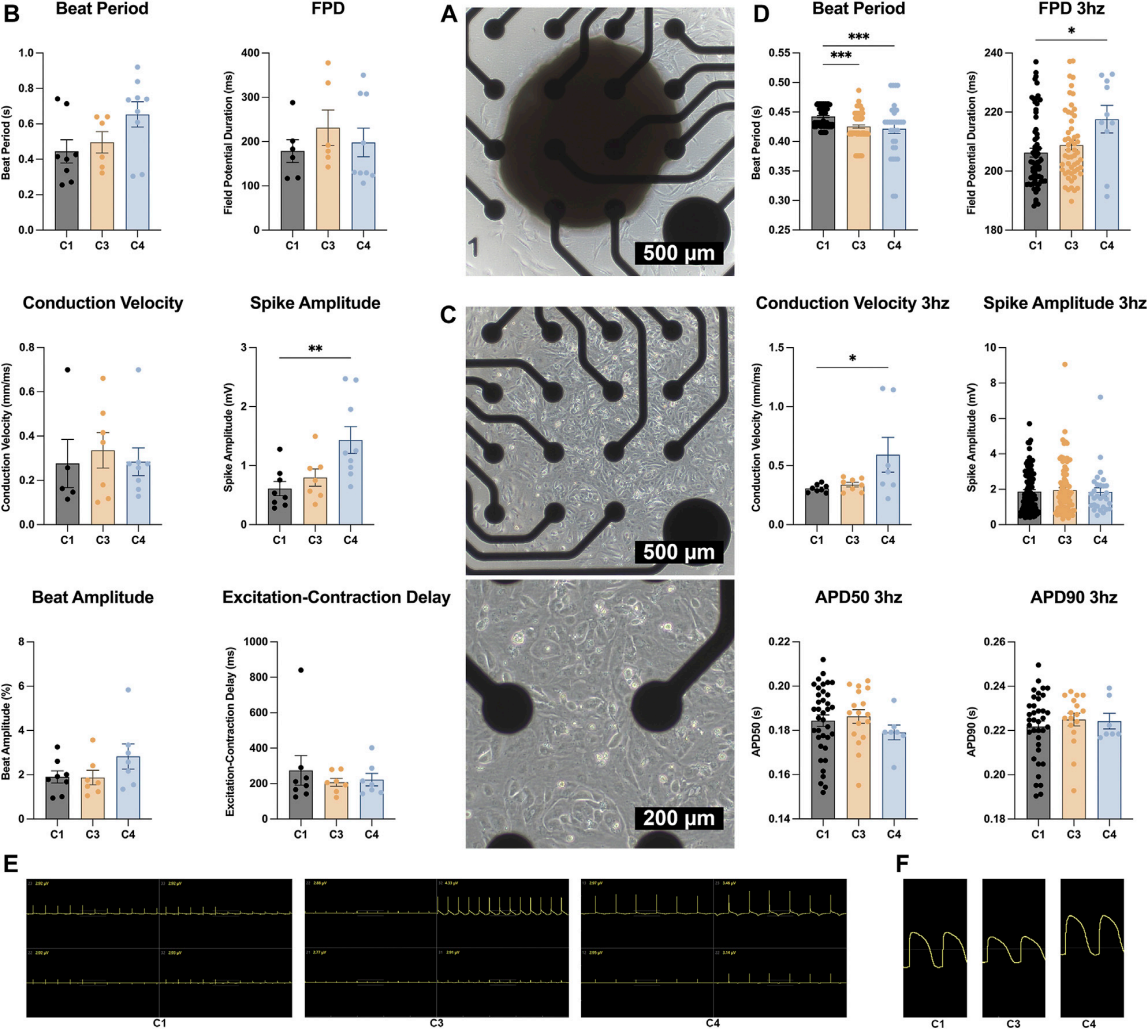
MEA assessments. **(A)** Whole C1, C3, and C4 spheroids (C1 black; C3 yellow; C4 blue) were attached to a 4 × 4 MEA; then, field potentials and impedance were measured and used to calculate **(B)** beat period, spike amplitude, field potential duration (FPD), conduction velocity, beat amplitude, and excitation. **(C)** C1, C3, and C4 spheroids (C1 black; C3 yellow; C4 blue) were dissociated on D30; then, CMs were collected and plated on the MEA electrodes. Field potentials and impedance were measured with and without pacing at 3 hz (330 ms or 180 bpm) and used to calculate **(D)** beat period, spike amplitude, FPD, conduction velocity, and the action potential duration until 50 and 90% recovery (APD50 and APD90), respectively. **(E)** MEA field potential traces for C1, C3, and C4 whole organoids only showing the 4 electrodes that were used for analysis. **(F)** MEA field potential traces of dissociated C1, C3, and C4 organoids after undergoing LEAP induction (*p < 0.05, **p < 0.01, ***p < 0.001; n > 6 per group).

MEA assessments conducted on D30 of whole spheroids (Figure 7A) indicated that the electromechanical properties of all three constructs were generally similar: spike amplitude measurements were significantly greater in C4 spheroids than in C1 spheroids, but variations among groups in beat period, beat amplitude, field-potential duration, conduction velocity, and excitation-contraction delay were small and not significant (Figure 7B). However, when CMs were isolated from the constructs (Figure 7C), beat period was significantly shorter for CMs from C3 or C4 spheroids (i.e., C3 or C4 CMs) than from C1 spheroids (C1 CMs), and field-potential durations were significantly longer in C4 than in C1 CMs when the cells were paced at 3 hz (Figure 7D). Conduction velocities were also significantly greater in C4 than C1 CMs during pacing, likely because the variability among measurements in C4 CMs was exceptionally high, while measurements of spike amplitudes and action-potential durations were similar in CMs from all three constructs.

## Discussion

The biological properties and activity of cells in the human heart are reproduced with greater fidelity by cardiac spheroids than by other *in-vitro* cell-culture systems. Thus, spheroids tend to provide a more accurate platform for modeling cardiac disease or drug development, and the individual cell populations present in cardiac spheroids may be more suitable for tissue engineering and other therapeutic applications. Here, we show that spheroids composed of hiPSC-derived CMs, ECs, SMCs, and CFs can be readily assembled and cultured for up to 60 days in solution; that C4 spheroids were significantly larger than C1 spheroids or C3 spheroids; and that the inclusion of vascular cells and CFs tended to promote sarcomere maturation and CM energy production. Furthermore, whereas apoptotic cells became increasingly common from D7 to D60 in C1 spheroids and C3 spheroids, the proportion in C4 spheroids remained stable throughout the culture period, which suggests that C4 spheroids are sufficiently durable for long-term studies. Notably, all four cardiac-cell types were differentiated from the same line of hiPSCs and, consequently, had the same genetic background, and the ratio of CMs, ECs, SMCs, and CFs (4:2:1:1) in C4 spheroids roughly followed the distribution trends found in human myocardium (Banerjee et al., 2007; Pinto et al., 2016; Gao et al., 2018; Arai et al., 2020; Beauchamp et al., 2020; Daly et al., 2021; Pretorius et al., 2021), but whether this ratio also produces the most native-like cardiac-cell phenotypes in cultured spheroids has yet to be conclusively demonstrated. The present study illustrates that additional optimization of this seeding ratio may be necessary to account for cell loss and restructuring that occurs and techniques for determining the composition in the fully matured tissue may be necessary. Additionally, the ideal ratio for different applications may vary depending on the region or state of myocardium that is being modeled.

The suspension culture system used in this report is broadly compatible with existing research equipment, and quality control checkpoints can be incorporated to maximize reproducibility; thus, our protocol can be readily adapted for a wide range of applications (Mattapally et al., 2018; LaBarge et al., 2019a; LaBarge et al., 2019b; Kim et al., 2020; Daly et al., 2021; Polonchuk et al., 2021) and production facilities (Abbasalizadeh et al., 2017; Adil and Schaffer, 2017; Li et al., 2017; Tomov et al., 2019). Furthermore, techniques for promoting spheroid fusion (Kim et al., 2018; Mattapally et al., 2018) and for the use of spheroids in 3D bioprinting (Duan, 2016; Maiullari et al., 2018; Aguilar et al., 2019; Alonzo et al., 2019; LaBarge et al., 2019b; Qasim et al., 2019; Lu Wang et al., 2021) have already been established, so the spheroids generated via this protocol could serve as building blocks for even larger and more sophisticated cardiac-tissue constructs, and because ECM production occurred spontaneously in C4 spheroids, exogenously administered ECM components may be unnecessary. However, only ∼50 spheroids per batch were produced in this study, and several of the steps were performed manually, so additional automation will be necessary to maximize productivity on an industrial scale.

The wide application of cardiac spheroid systems has led many groups to develop a range of models for both *in vitro* and *in vivo* uses. The microtissues in the present study achieved a greater overall diameter (500–1000 µm) than were previously produced (50–400 µm) with maintained cellular complexity and viability (Beauchamp et al., 2020; Giacomelli et al., 2020; Giacomelli et al., 2021). Although decreased central necrosis was observed in the C4 spheroids, the large diameter and fusion process raises concerns for nutrient and oxygen diffusion (Langan et al., 2016) which will require further assessment in future studies. The use of bioreactor culture environment rather than static culture may provide partial mitigation. Spheroids were also shown to maintain beating and viability over a 60 days period which provides greater longitudinal analysis than the single time points at 28, 20, 15, 30, and 12 days used by others (Ravenscroft et al., 2016; Giacomelli et al., 2017; Israeli et al., 2020; Polonchuk et al., 2021; Thomas et al., 2021). Finally, the protocol of fusing the multiple cell types into larger cardiac spheroids described here uses 96 well plates, a widely used format, and doesn’t require CM dissociation, a challenging and labor intensive step, while still maintaining consistent ratios of cell types. This is in comparison to complicated fabrication processes involving complete CM dissociation, hanging drop methods, agarose mold production, and additional centrifugation steps (Nugraha et al., 2019; Buono et al., 2020; Kupfer et al., 2020; Giacomelli et al., 2021; Thomas et al., 2021) and high variability due to spontaneous differentiation compared with the combination of purified cell types (Hoang et al., 2018; Schulze et al., 2019; Israeli et al., 2020) found in other studies.

Although the current study showed improvements in key biomolecular and functional markers a limitation in the data was the lack of functional cardiac metrics at each time point. Particularly, the lack of electromechanical testing at D60 when other biomarkers appeared to show the greatest improvement leaves a gap in maturation characterization. Future iterations of the cardiac spheroid model should consider incorporating data from both early and late time points for all analyses to provide greater longitudinal understanding. Additionally, the media composition chosen was based on previous work with cardiac cell coculture (Giacomelli et al., 2017; Gao et al., 2018; Noor et al., 2019; Pretorius et al., 2021) but was not optimized or fully defined. The use of FBS, although routine, has previously been shown to lead to dedifferentiation of ECs (Kaupisch et al., 2012; Sriram et al., 2015) and an alternative media formulation may be considered for future model iterations that is cGMP compliant (Zimmermann, 2020).

In conclusion, the experiments in this report demonstrate that C4 spheroids composed of four different hiPSC-derived cell populations (CMs, ECs, SMCs, and CFs) can be efficiently manufactured via a 3D suspension culture that is compatible with a wide range of applications and research equipment. Measures of spheroid size and cell viability were significantly greater in C4 spheroids than in spheroids lacking CFs after 60 days of culture, and the inclusion of vascular cells and CFs tended to promote sarcomere maturation and CM energy production. Future work will continue to investigate methods for improving CM maturity and spheroid yield while incorporating automated processes that are suitable for large-scale production facilities (Table 1).

**TABLE 1:** Data collected from MEA analysis of whole and dissociated spheroids.

Format	Group	Beat period (s)	Spike amplitude (mV)	FPD (ms)	Conduction velocity (cm/s)	Beat amplitude (%)	ECD (ms)	APD50 (ms)	APD90 (ms)
Whole Spheroid	C1	0.45 ± 0.07	0.61 ± 0.12	179 ± 26	27.6 ± 10.9	1.90 ± 0.27	275 ± 83		
	C3	0.50 ± 0.06	0.80 ± 0.15	231 ± 40	33.6 ± 8.0	1.88 ± 0.33	207 ± 22		
	C4	0.65 ± 0.07	1.43 ± 0.23	198 ± 32	28.5 ± 6.3	2.83 ± 0.57	222 ± 35		
Isolated CMs (Paced at 3 Hz)	C1	0.44 ± 0.01	1.87 ± 0.12	206 ± 2	30.7 ± 1.1	2.02 ± 0.13	142 ± 3	184 ± 3	222 ± 2
	C3	0.43 ± 0.01	1.95 ± 0.15	209 ± 2	34.0 ± 1.9	2.10 ± 0.19	144 ± 3	186 ± 3	225 ± 3
	C4	0.42 ± 0.01	1.85 ± 0.24	218 ± 5	59.3 ± 14.7	1.74 ± 0.17	146 ± 3	179 ± 3	224 ± 4

## Data Availability

The raw data supporting the conclusion of this article will be made available by the authors, without undue reservation.
