# Supplementary material for: A three-dimensional culture system for generating cardiac spheroids composed of cardiomyocytes, endothelial cells, smooth-muscle cells, and cardiac fibroblasts derived from human induced-pluripotent stem cells
Source: Front Bioeng Biotechnol. 2022 Jul 22;10:908848. doi: 10.3389/fbioe.2022.908848 (PMC9361017; doi:10.3389/fbioe.2022.908848)
Supplement: Supplementary file 1 [file DataSheet1.PDF]

Supplementary Material

1 Supplementary Figures

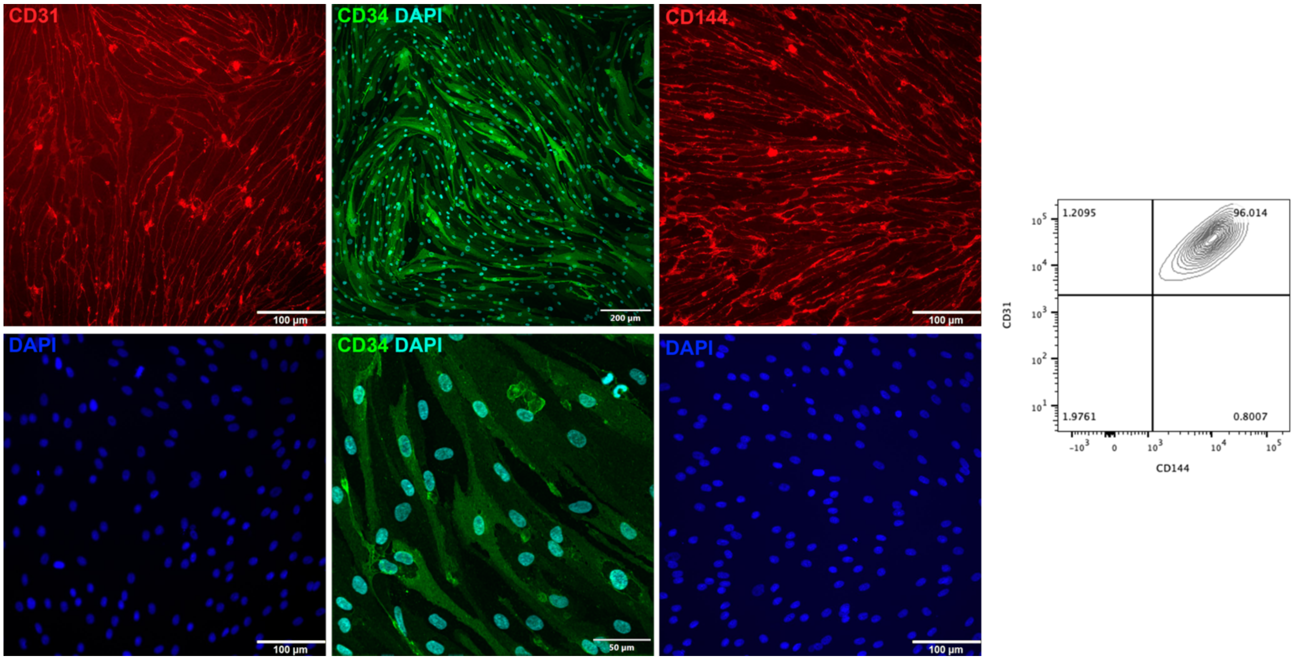

Supplementary Figure S1. Characterization data from iPSC-ECs illustrating their purity and expression of key markers.

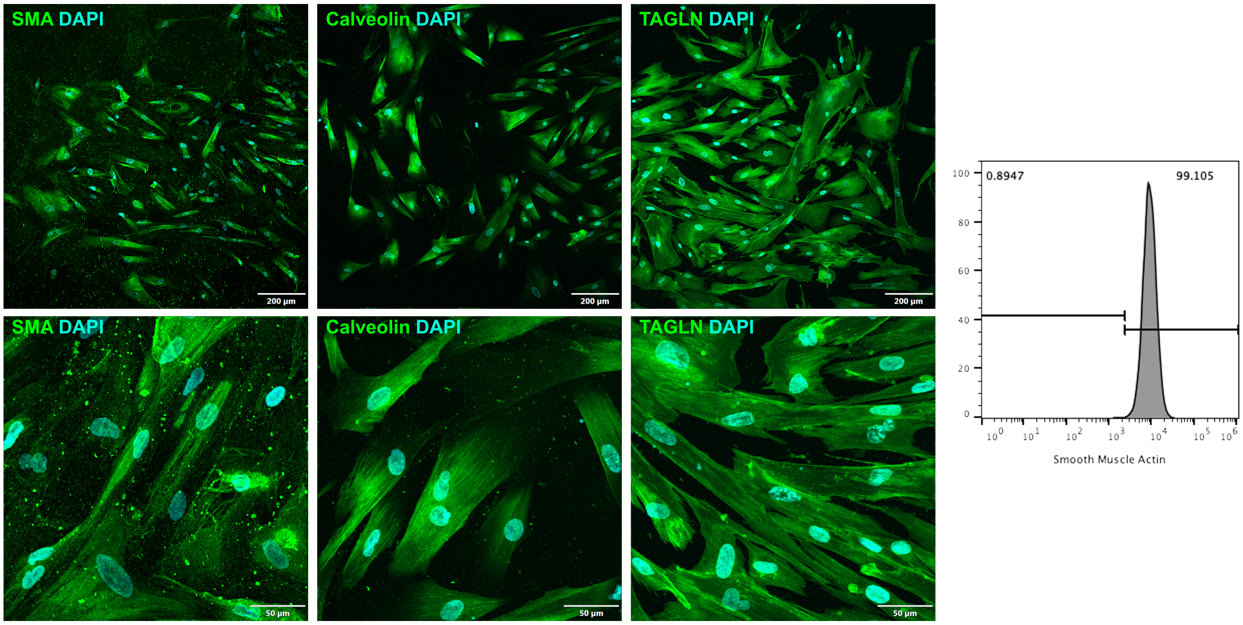

Supplementary Figure S2. Characterization data from iPSC-SMCs illustrating their purity and expression of key markers.

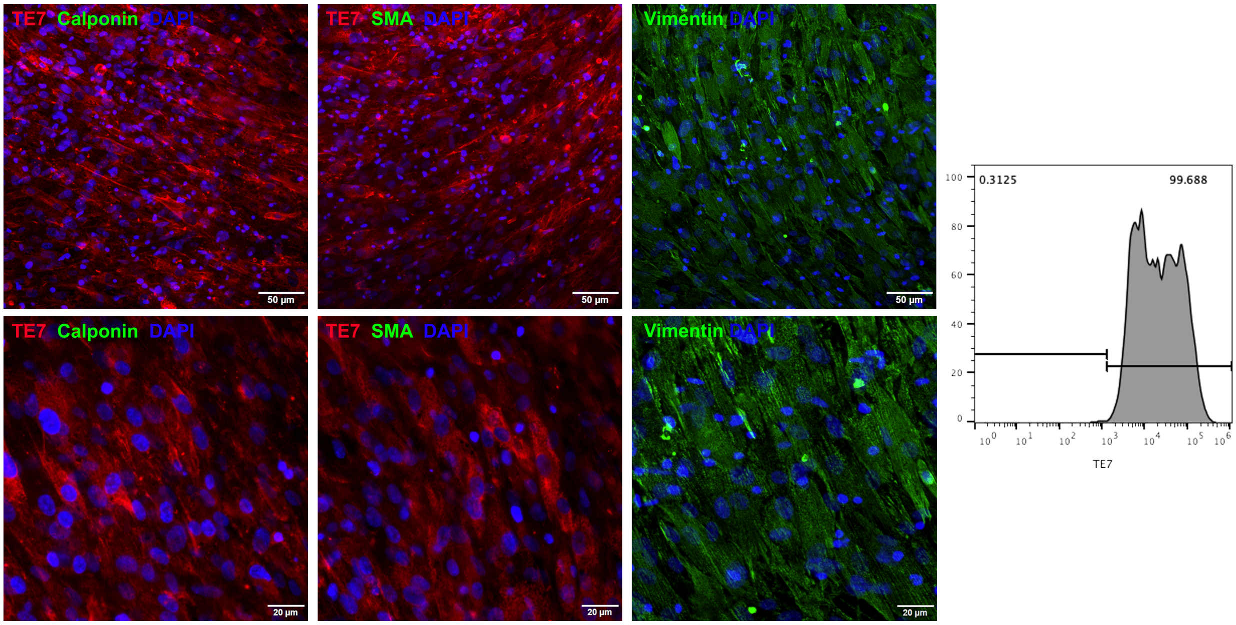

Supplementary Figure S3. Characterization data from iPSC-CFs illustrating their purity and expression of key markers.

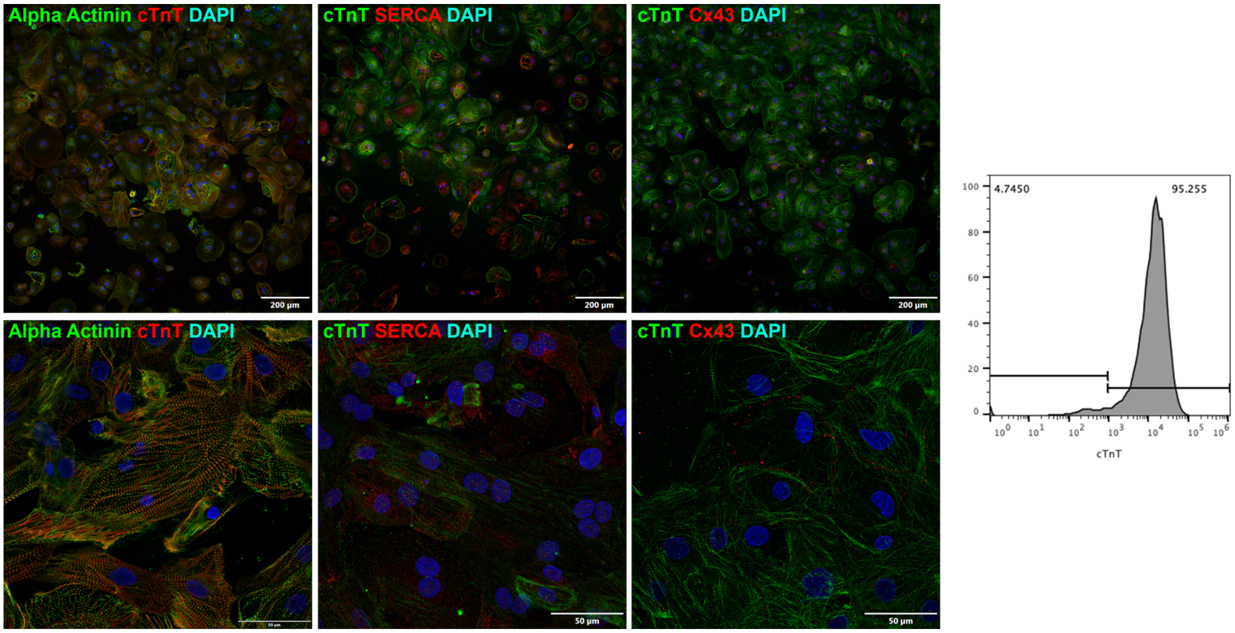

Supplementary Figure S4. Characterization data from iPSC-CM spheroids after dissociation illustrating their purity and expression of key markers.

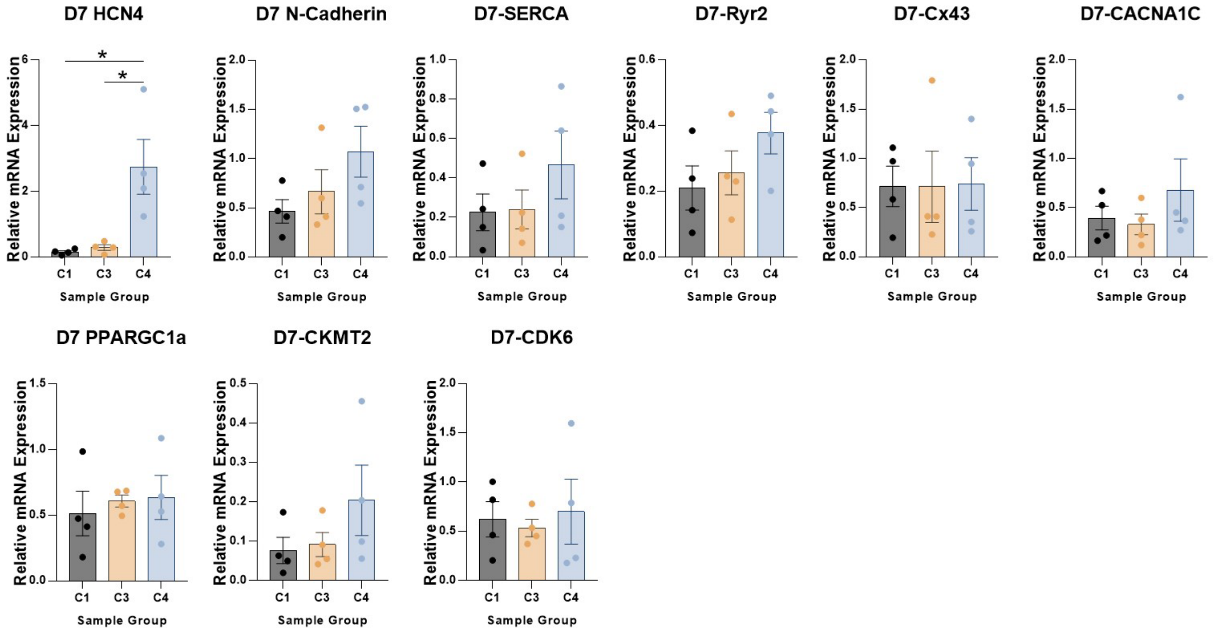

Supplementary Figure S5. The magnitude of expression for genes that contribute to CM (A) electrical conduction (HCN4, N-cadherin, SERCA, Ryr2, Cx43, CACNA1C), (B) metabolism (PPARGC1a, CKMT2), and (C) cell-cycle activity (CDK6) was evaluated in C1, C3, and C4 spheroids on D7 (C1 black; C3 yellow; C4 blue) via qPCR; measurements were normalized to intrinsic GAPDH mRNA abundance (*p<0.05; n>4 per group).

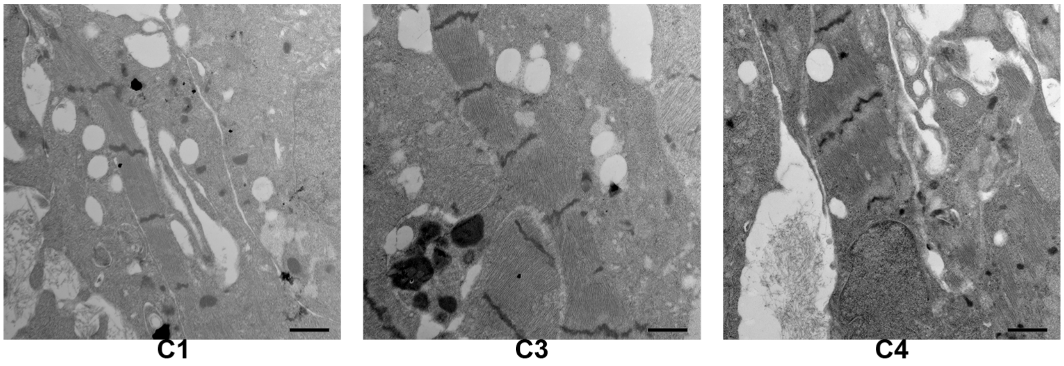

Supplementary Figure S6. Whole C1, C3, and C4 spheroids on day 60 were sectioned and imaged via TEM (bar=1 μM)

2 Supplementary Tables

2.1 Table S1: Antibodies used in this study along with their sourcing and dilutions

Antibody Name	Source	Catalog Number	RRID	Application (FC=Flow Cytometry, I=Immunostaining, WB= Western Blot)	Dilution
Troponin T, Cardiac Isoform Ab-1, Mouse Monoclonal Antibody	Thermo Fisher	MS295P	AB_61806	FC	1:200
Zenon™ Mouse IgG_1 Labeling Kit	Invitrogen	Z-25002	AB_2736941	FC	N/A
Alexa Fluor® 647 Anti-CD31 antibody [JC/70A]	Abcam	ab215912	AB_2890260	I	1:200
Alexa Fluor® 647 Mouse Anti-Human CD31	BD Bioscience	561654	AB_10896969	FC	1:20
PE Mouse Anti-Human CD34	BD Bioscience	550761	AB_393871	I	1:5
PE Mouse anti-Human CD144	BD Bioscience	560410	AB_1645502	I, FC	1:5
Anti-alpha smooth muscle Actin antibody	Abcam	ab5694	AB_2223021	I, FC	1:200, 1:100
Anti-Caveolin-3 antibody	Abcam	ab2912	AB_2291095	I	1:200
Anti-TAGLN/Transgelin antibody	Abcam	ab14106	AB_443021	I	1:200
Anti-Fibroblasts Antibody, clone TE-7	Sigma	CBL271	AB_93449	I, FC	1:200
Recombinant Anti-Vimentin antibody [EPR3776]	Abcam	ab92547	AB_10562134	I	1:200
Anti-SOX2 antibody	Abcam	ab97959	AB_2341193	FC	1:100
Anti-SSEA4 antibody [MC813-70]	Abcam	ab16287	AB_778073	FC	1:100
Anti-TRA-1-60 (R) antibody [TRA-1-60]	Abcam	ab16288	AB_778563	FC	1:100
Recombinant Anti-Cardiac Troponin T antibody [EPR3695]	Abcam	ab91605	AB_2050427	I	1:200
Monoclonal Anti-α-Actinin (Sarcomeric)	Sigma	A7811	AB_476766	I	1:200
Anti-Cardiac Troponin T antibody [1F11]	Abcam	ab10214	AB_2206574	I	1:200
Anti-SERCA2 ATPase antibody	Abcam	ab3625	AB_303961	I	1:200
Anti-Connexin 43 / GJA1 antibody - Intercellular Junction Marker	Abcam	ab11370	AB_297976	I	1:200
Anti-Troponin I Type 3 Rabbit Polyclonal Antibody	Proteintech	21652-1-AP	AB_2878898	WB	1:600
Anti-Troponin I Type 1 Rabbit Polyclonal Antibody	Proteintech	16102-1-AP	AB_2206103	WB	1:1000
Anti-MYH6 Rabbit Polyclonal Antibody	Proteintech	22281-1-AP	AB_2736822	WB	1:600
MYH7 Rabbit anti-Human, Mouse	Proteintech	22280-1-AP	AB_2736821	WB	1:1000
Myosin Light Chain 2/MLC-2V Polyclonal antibody	Proteintech	10906-1-AP	AB_2147453	WB	1:1000
MYL7 Polyclonal antibody	Proteintech	17283-1-AP	AB_2250998	WB	1:1000
β-Actin Antibody	Cell Signaling	4967S	AB_330288	WB	1:1000
In Situ Cell Death Detection Kit, TMR red	Sigma	12156792910	N/A	I	N/A
Goat anti-Mouse IgG (H+L) Highly Cross-Adsorbed Secondary Antibody, Alexa Fluor Plus 488	Invitrogen	A32723	AB_2633275	Secondary Antibody	1:200

Goat anti-Rabbit IgG (H+L) Highly Cross-Adsorbed Secondary Antibody, Alexa Fluor Plus 488	Invitrogen	A32731	AB_2633280	Secondary Antibody	1:200
Goat anti-Rabbit IgG (H+L) Highly Cross-Adsorbed Secondary Antibody, Alexa Fluor Plus 555	Invitrogen	A32732	AB_2633281	Secondary Antibody	1:200

2.2 **Table S2.** qPCR primers used for quantifying gene expression levels in this study.

**Target Name**	**Forward Primer**	**Reverse Primer**
SOX2	GAGGGCTGGACTGCGAACT	TTTGCACCCCTCCCAATTC
OCT4	CAGTGCCCGAAACCCACAC	GGAGACCCAGCAGCCTCAAA
Nanog	TTTGGAAGCTGCTGGGGAAG	GATGGGAGGAGGGGAGAGGA
Alpha-MHC	CTCCGTGAAGGGATAACCAGG	TTCACAGTCACCGTCTTCCC
Beta-MHC	ACCAACCTGTCCAAGTTCCG	TCATTCAAGCCCTTCGTGCC
MLC-2a	GGAGTTCAAAGAAGCCTTCAGC	AAAGAGCGTGAGGAAGACGG
MLC-2v	ACATCATCACCCACGGAGAAGAGA	ATTGGAACATGGCCTCTGGATGGA
TNNI 1	GGTGGATGAGGAGCGATACG	GCTTCAGGTCCTTAATCTCCCTG
TNNI 3	GGAGGACACCGAGAAGGAAAAC	TCAAACTTTTTCTTGCGGCCC
PPARGC1A	GCTTTCTGGGTGGACTCAAGT	GAGGGCAATCCGTCTTCATCC
CKMT2	GCTCCGGCTTCAAGACACTC	TGCGCTTGGAGGAAATAGCC
HCN4	CCCGGAGGCCGAGGT	TCAGGTCCCAGTAAAATCTGAAGTC
SERCA	TCACCTGTGAGAATTGACTGG	AGAAAGAGTGTGCAGCGGAT
RyR2	TTGGAAGTGGACTCCAAGAAA	CGAAGACGAGATCCAGTTCC
Cx43	GGTGACTGGAGCGCCTTAG	GCGCACATGAGAGATTGGGA
N-Cadherin	AGCCAACCTTAACTGAGGAGT	GGCAAGTTGATTGGAGGGATG
CACNA1C	TGATTCCAACGCCACCAATTC	GAGGAGTCCATAGGCGATTACT
CDK6	GACTGACACTCGCAGCCC	CAGTCCAGAATCATTGCACCTGAG
CD31	TCAGACGTGCAGTACACGGA	GGGAGCCTTCCGTTCTAGAGT
Alpha-SMA	TATCCCCGGGACTAAGACGG	CACCATCACCCCCTGATGTC
FAP	AGGGATGGTCATTGCCTTGG	ATCCTCCATAGGACCAGCCC
GAPDH	GTGGACCTGACCTGCCGTCT	GGAGGAGTGGGTGTCGCTGT

2.3 **Table S3.** Composition of organoid media and component sourcing for 50 mL total volume.

**Component Name**	**Volume**	**Concentration**	**Sourcing**
DMEM/F-12 with GlutaMAX	47.5 mL		Thermo Scientific Cat# 10565042
B27 Supplement	1 mL	2%	Fisher Scientific Cat# 17-504-044
FBS	1 mL	2%	Thermo Fisher Scientific Cat# A4736301
VEGF (50ng/µL)	25 µL	25 ng/mL	Fisher Scientific Cat# PHC9393
FGF (5 ng/µL)	25 µL	2.5 ng/mL	R&D Systems Cat# 3718-FB-100
Penicillin-Streptomycin	500 µL	1x	Fisher Scientific Cat# 15140122

3 imageJ Plugins

3.1 Organoid Size Analysis

```
//This macro aims to automate spheroid size measurement in three-dimensional cell culture. It requires input and output folders with images only, processes the images, records a file with spheroid measurements (Area, Ferret max, Ferret min, etc.) and writes an image with the outline/s of the determined spheroid/s.
//The spheroid detection and size determination function to be repeated for every image is defined below
function action(inputFolder,outputFolder,filename) {
open(inputFolder + filename);
//sets scale to predetermined values from calibration slide
run("Set Scale...", "distance=1.1801 known=1 unit=µm global");
run("16-bit");
//run("Brightness/Contrast...");
run("Enhance Contrast", "saturated=0.35");
//Uses Yen thresholding algorythm
setAutoThreshold("Mean");
//Li is the alternative
//Yen Alternative
//Moments Alternative
setOption("BlackBackground", false);
run("Convert to Mask");
//Gets the ratio between black (spheroid) and white (background) pixels. If we assume a single spheroid, the ratio between black and white pixels would allow us to estimate the size of the spheroid.
getHistogram(0,hist,256);
ratio = hist[255]/hist[0];//0.002; //
//If there are more pixels detected as spheroid(black) than background(white) then the spheroid has not been detected due to variations in background
if (ratio>1) {
	// closes the image, reopens it, subtracts the background and proceeds as normal
	close();
	open(inputFolder + filename);
	run("16-bit");
	// Subtract Background is not used in the default function because it can lead to merging of spheroids and debris or it can remove the core of the spheroid leaving a very thin interrupted edge. In certain cases where the edges of a spheroid are very bright removing the background can give better results.
	run("Subtract Background...", "rolling=50 light");
	setAutoThreshold("Mean");
	setOption("BlackBackground", false);
	run("Convert to Mask");
	run("Remove Outliers...", "radius=15 threshold=0 which=Dark");
	getHistogram(0,hist,256);
	ratio = hist[255]/hist[0];};

	//The strategy here is to act differently according to spheroid size. The general pattern is to expand and then shrink back the spheroids in order to include all cells on the edges. Then a series of functions are used to remove noise and the Watershed function separates fused or superimposed particles. The Analyze particles function is targeted to the specific spheroid size according to the black/white pixel ratio.
if (ratio<0.001) {
	run("Maximum...", "radius=8");
	run("Fill Holes");
	run("Minimum...", "radius=8");
	//small spheroids require a more "gentle" function to clean up noise
	run("Median...", "radius=2");
	run("Maximum...", "radius=25");
	run("Minimum...", "radius=25");
	run("Fill Holes");
	run("Watershed");
	run("Analyze Particles...", "size=4000-Infinity circularity=0.80-1.00 show=[Overlay Outlines] display exclude include summarize");};
```

```
if (ratio >=0.001 && ratio<0.01) {
		run("Maximum...", "radius=8");
		run("Fill Holes");
		run("Minimum...", "radius=8");
		//slightly bigger spheroids and a more rigorous function to remove noise
		run("Remove Outliers...", "radius=10 threshold=0 which=Dark");
		run("Watershed");
		run("Analyze Particles...", "size=1000-Infinity circularity=0.80-1.00 show=[Overlay Outlines] display exclude
include summarize");};

if (ratio>=0.01 && ratio<0.2) {
		run("Maximum...", "radius=8");
		run("Fill Holes");
		run("Minimum...", "radius=8");
		run("Remove Outliers...", "radius=15 threshold=0 which=Dark");
		run("Median...", "radius=4");
		run("Watershed");
		run("Analyze Particles...", "size=20000-Infinity circularity=0.20-1.00 show=[Overlay Outlines] display exclude
include summarize");};

if (ratio>=0.2 && ratio<1) {
		//Very big spheroids generally do not need to be expanded much to fill up the edges.
		run("Maximum...", "radius=3");
		run("Fill Holes");
		run("Minimum...", "radius=3");
		//Outliers and noise are removed rigorously
		run("Remove Outliers...", "radius=50 threshold=0 which=Dark");
		run("Minimum...", "radius=30");
		run("Maximum...", "radius=30");
		run("Watershed");
		run("Analyze Particles...", "size=50000-Infinity circularity=0.20-1.00 show=[Overlay Outlines] display exclude
include summarize");};

 if (Overlay.size > 0) {
//Sends particles detected to the ROI manager
run("To ROI Manager");
close();
//Reopens the original image and pastes the outlines of the determined particles onto it
open(inputFolder + filename);
run("From ROI Manager");
outputPath = outputFolder + filename;
save(outputPath);
close(); }
else {
		close();
};
call("java.lang.System.gc");
};
call("java.lang.System.gc");
run("Clear Results");
inputFolder = getDirectory("Choose the input folder!");
outputFolder = getDirectory("Choose the output folder!");
//Delete the next line if you want to see how the macro works on the images. However that will reduce processing speed.
setBatchMode(true);
images = getFileList(inputFolder);
//Sets the measurements that are recorded for each spheroid
run("Set Measurements...", "area centroid shape feret's display add redirect=None decimal=3");
//That is the cycle that runs through all images
```

```
for (i=0; i<images.length; i++) {
 action(inputFolder,outputFolder,images[i]);
 showProgress(i, images.length);
};
//Writes in the Results and Summary windows and saves the data.
selectWindow("Results");
saveAs("Measurements", "" + outputFolder + "Results.txt");
selectWindow("Summary");
saveAs("Text", "" + outputFolder +"Summary.txt");
setBatchMode(true);
```

3.2 TUNEL nuclei analysis

```
//This macro aims to automate TUNEL nuclei counting in immunofluorescent stained sections. It requires input and output folders with images only, processes the images, records a file with DAPI and TUNEL area measurements and writes an image with the quantified regions.
function action(inputFolderDAPI,inputFolderTUNEL,outputFolder,filename1, filename2) {
open(inputFolderDAPI + filename1);
//sets scale to predetermined values from calibration slide
run("Set Scale...", "distance=160 known=200 unit=µm global");
run("16-bit");

//run("Brightness/Contrast...");
run("Enhance Contrast", "saturated=0.35");
//Uses Li thresholding algorythm
setAutoThreshold("Li");

setOption("BlackBackground", false);
run("Convert to Mask");
run("Invert");
run("Canvas Size...", "width=1024 height=950 position=Top-Center");
run("Create Selection");
run("Measure");
outputPath = outputFolder + filename1;
save(outputPath);

open(inputFolderTUNEL + filename2);
//sets scale to predetermined values from calibration slide
run("Set Scale...", "distance=160 known=200 unit=µm global");
run("16-bit");

//run("Brightness/Contrast...");
run("Enhance Contrast", "saturated=0.35");

//Uses Li thresholding algorythm
setAutoThreshold("Li");

setOption("BlackBackground", false);
run("Convert to Mask");
run("Invert");
run("Canvas Size...", "width=1024 height=950 position=Top-Center");

imageCalculator("Min create", filename1, filename2);
run("Create Selection");
```

```
run("Measure");

outputPath = outputFolder + filename2;
save(outputPath);
close();

call("java.lang.System.gc");
};
call("java.lang.System.gc");
run("Clear Results");
inputFolderDAPI = getDirectory("Choose the DAPI input folder!");
inputFolderTUNEL = getDirectory("Choose the TUNEL input folder!");
outputFolder = getDirectory("Choose the output folder!");
//Delete the next line if you want to see how the macro works on the images. However that will reduce processing speed.
setBatchMode(true);
imagesDAPI = getFileList(inputFolderDAPI);
imagesTUNEL = getFileList(inputFolderTUNEL);
//Sets the measurements that are recorded for each spheroid
run("Set Measurements...", "area display add redirect=None decimal=3");
//That is the cycle that runs through all images
for (i=0; i<imagesDAPI.length; i++) {
 action(inputFolderDAPI,inputFolderTUNEL,outputFolder,imagesDAPI[i],imagesTUNEL[i]);
 showProgress(i, imagesDAPI.length);
};
//Writes in the Results and Summary windows and saves the data.
selectWindow("Results");
saveAs("Measurements", "" + outputFolder + "Results.txt");
//selectWindow("Summary");
//saveAs("Text", "" + outputFolder +"Summary.txt");
setBatchMode(true);
```

3.3 Cell Coverage Analysis

```
//This macro aims to automate cell coverage area in immunofluorescent stained sections. It requires input and output folders with images only, processes the images, records a file with cTnT, CD31, TE7 and whole area measurements and writes an image with the quantified regions in white.

function action(cTnT,CD31,TE7,Whole,outputFolder,CM,EC,CF,organoid) {
open(Whole + organoid);
//sets scale to predetermined values from calibration slide
run("Set Scale...", "distance=1 known=1 unit=µm global");
run("16-bit");

//run("Brightness/Contrast...");
run("Enhance Contrast", "saturated=0.35");
//Uses Li thresholding algorythm
setAutoThreshold("Li");

setOption("BlackBackground", false);
run("Convert to Mask");
run("Despeckle");
run("Invert");
run("Canvas Size...", "width=1024 height=950 position=Top-Center");
run("Create Selection");
run("Measure");
```

```
outputORGANOID = outputFolder + organoid;
save(outputORGANOID);

open(CD31 + EC);
//sets scale to predetermined values from calibration slide
run("Set Scale...", "distance=1 known=1 unit=µm global");
run("16-bit");

//run("Brightness/Contrast...");
run("Enhance Contrast", "saturated=0.35");
//Uses Li thresholding algorythm
setAutoThreshold("Li");

setOption("BlackBackground", false);
run("Convert to Mask");
run("Despeckle");
run("Invert");
run("Canvas Size...", "width=1024 height=950 position=Top-Center");
imageCalculator("Subtract", organoid, EC);
run("Create Selection");
run("Measure");

outputEC = outputFolder + EC;
save(outputEC);

open(TE7 + CF);
//sets scale to predetermined values from calibration slide
run("Set Scale...", "distance=1 known=1 unit=µm global");
run("16-bit");

//run("Brightness/Contrast...");
run("Enhance Contrast", "saturated=0.35");
//Uses Li thresholding algorythm
setAutoThreshold("Li");

setOption("BlackBackground", false);
run("Convert to Mask");
run("Despeckle");
run("Invert");
run("Canvas Size...", "width=1024 height=950 position=Top-Center");
imageCalculator("Subtract", organoid, CF);
run("Create Selection");
run("Measure");

outputCF = outputFolder + CF;
save(outputCF);

open(cTnT + CM);
//sets scale to predetermined values from calibration slide
run("Set Scale...", "distance=1 known=1 unit=µm global");
run("16-bit");

//run("Brightness/Contrast...");
run("Enhance Contrast", "saturated=0.35");
//Uses Li thresholding algorythm
setAutoThreshold("Li");
```

```
setOption("BlackBackground", false);
run("Convert to Mask");
run("Despeckle");
run("Invert");
run("Canvas Size...", "width=1024 height=950 position=Top-Center");
imageCalculator("Subtract", organoid, CM);
run("Create Selection");
run("Measure");

outputCM = outputFolder + CM;
save(outputCM);
close();

call("java.lang.System.gc");
};
call("java.lang.System.gc");
run("Clear Results");
cTnT = getDirectory("Choose the cTnT folder!");
CD31 = getDirectory("Choose the CD31 folder!");
TE7 = getDirectory("Choose the TE7 folder!");
Whole = getDirectory("Choose the Whole folder!");
outputFolder = getDirectory("Choose the output folder!");
//Delete the next line if you want to see how the macro works on the images. However that will reduce processing speed.
setBatchMode(true);

icTnT = getFileList(cTnT);
iCD31 = getFileList(CD31);
iTE7 = getFileList(TE7);
iWHole = getFileList(Whole);

//Sets the measurements that are recorded for each spheroid
run("Set Measurements...", "area display add redirect=None decimal=3");
//That is the cycle that runs through all images
for (i=0; i<icTnT.length; i++) {
 action(cTnT,CD31,TE7,Whole,outputFolder,icTnT[i],iCD31[i],iTE7[i],iWHole[i]);
 showProgress(i, cTnT.length);
};
//Writes in the Results and Summary windows and saves the data.
selectWindow("Results");
saveAs("Measurements", "" + outputFolder + "Results.txt");
//selectWindow("Summary");
//saveAs("Text", "" + outputFolder +"Summary.txt");
setBatchMode(true);
```
